# 完全电视胸腔镜手术与胸腔镜辅助小切口手术治疗早期非小细胞肺癌临床疗效的*Meta*分析

**DOI:** 10.3779/j.issn.1009-3419.2017.05.02

**Published:** 2017-05-20

**Authors:** 兵 王, 方 律, 亮 赵, 闽军 杜, 树庚 高

**Affiliations:** 100021 北京, 国家癌症中心/中国医学科学院北京协和医学院肿瘤医院胸外科 Department of Toracic Surgical Oncology, National Cancer Center/Cancer Hospital, Chinese Academy of Medical Sciences and Peking Union Medical College, Beijing 100021, China

**Keywords:** 肺肿瘤, 全电视胸腔镜手术, 胸腔镜辅助小切口直视下手术, *Meta*分析, Lung neoplasms, VATS, VAMT, *Meta*-analysis

## Abstract

**背景与目的:**

目前未见对全电视胸腔镜手术与胸腔镜辅助小切口手术治疗非小细胞肺癌临床疗效和安全性的系统评价。在本*meta*分析中，我们比较两组手术治疗非小细胞肺癌的临床效果。

**方法:**

系统地检索7个主要的医学期刊数据库：PubMed、Embase、CNKI、VIP、ISI Web of Science、the Cochrane Library和CBM，收集时间涵盖2006年5月-2016年5月的有关完全电视胸腔镜手术与胸腔镜辅助小切口手术治疗非小细胞肺癌的随机对照研究（randomized controlled trials, RCT）。由两名研究者对文献质量独立进行评价并提取相关数据，采用RevMan 5.3软件进行*meta*分析。

**结果:**

共纳入13篇RCT，共1, 605例患者。其中完全电视胸腔镜组（video-assisted thoracoscopic surgery, VATS）815例，胸腔镜辅助小切口组（video-assisted mini-thoracotomy, VAMT）790例。*Meta*分析结果显示：全胸腔镜手术与胸腔镜辅助小切口手术治疗非小细胞肺癌相比，手术时间（SMD=13.56, 95%CI: 4.96-22.16）、术中出血量（SMD=-33.68, 95%CI: -45.70--21.66）、胸腔引流管留置时间（SMD=-1.05, 95%CI: -1.48--0.62）、胸腔引流量（SMD=-83.69, 95%CI: -143.33--24.05）、术后疼痛程度（SMD=-1.68, 95%CI: -1.98--1.38）、术后住院时间（SMD=-2.27, 95%CI: -3.23--1.31）、两组淋巴结清扫数目（SMD=-0.48, 95%CI: -0.80--0.17）等方面的差异有统计学意义；而两组术中的并发症发生率（SMD=0.83, 95%CI: 0.54-1.29）、手术死亡率（SMD=0.95, 95%CI: 0.55-1.63）和1年复发率（RR=0.87, 95%CI: 0.34-2.24）差异无统计学意义（*P*＞0.05）。

**结论:**

完全电视胸腔镜手术与胸腔镜辅助小切口切除术相比，治疗非小细胞肺癌的并发症发生率和手术死亡率相当，但二者在淋巴结清扫数目、手术时间、术中出血量、胸腔引流量、胸腔引流管留置时间、术后疼痛程度和术后住院时间等方面有差异。现有的临床资料显示：相比胸腔镜辅助小切口手术，全胸腔镜手术是治疗早期非小细胞肺癌更好的选择。

## Abstract (en)

**Background and objective:**

The aim of this study is to assess the effect of video-assisted thoracoscopic surgery (VATS) and video-assisted mini-thoracotomy (VAMT) in the treatment of non-small cell lung cancer (NSCLC).

**Methods:**

We searched PubMed, EMbase, CNKI, VIP and ISI Web of Science to collect randomized controlled trials (RCTs) of VATS versus VAMT for NSCLC. Each database was searched from May 2006 to May 2016. Two reviewers independently assessed the quality of the included studies and extracted relevant data, using RevMan 5.3 *meta*-analysis software.

**Results:**

We finally identified 13 RCTs involving 1, 605 patients. There were 815 patients in the VATS group and 790 patients in the VAMT group. The results of *meta*-analysis were as follows: statistically significant difference was found in the harvested lymph nodes (SMD=-0.48, 95%CI: -0.80--0.17), operating time (SMD=13.56, 95%CI: 4.96-22.16), operation bleeding volume (SMD=-33.68, 95%CI: -45.70--21.66), chest tube placement time (SMD=-1.05, 95%CI: -1.48--0.62), chest tube drainage flow (SMD=-83.69, 95%CI: -143.33--24.05), postoperative pain scores (SMD=-1.68, 95%CI: -1.98--1.38) and postoperative hospital stay (SMD=-2.27, 95%CI: -3.23--1.31). No statistically significant difference was found in postoperative complications (SMD=0.83, 95%CI: 0.54-1.29) and postoperative mortality (SMD=0.95, 95%CI: 0.55-1.63) between videoassisted thoracoscopic surgery lobectomy and video-assisted mini-thoracotomy lobectomy in the treatment of NSCLC.

**Conclusion:**

Compared with video-assisted mini-thoracotomy lobectomy in the treatment of non-small cell lung cancer, the amount of postoperative complications and postoperative mortality were almost the same in video-assisted thoracoscopic lobectomy, but the amount of harvested lymph nodes, operating time, blood loss, chest tube drainage flow, and postoperative hospital stay were different. VATS is safe and effective in the treatment of NSCLC.

目前，肺癌在全世界恶性肿瘤中已居首位，其发病率和死亡率非常高，且有逐年增加的趋势^[1, 2]^。非小细胞肺癌（non-small cell lung cancer, NSCLC）是肺癌的一种，占全部肺癌的80%-85% ^[3]^。

微创手术因手术创伤小、术后疼痛轻、恢复快等优越性，成为目前外科手术的研究热点。胸腔镜是胸部微创外科的代表性手术。从传统的开放手术，到为减少损伤选择不同手术切口，逐步进展到小切口，腔镜辅助下的小切口，最后发展到全胸腔镜手术，为更多患者解除痛苦的梦想成为现实。目前临床微创手术中最常用的两种手术方式为完全电视胸腔镜手术（video-assisted thoracoscopic surgery, VATS）和胸腔镜辅助小切口直视下手术（video-assisted mini-thoracotomy, VAMT）。

到目前为止，虽有一些散在的研究比较了二者的临床效果，但尚无一致的结论。未见有关全电视胸腔镜与胸腔镜辅助小切口手术治疗NSCLC的系统评价报道。因此，为对VATS和VAMT治疗NSCLC的疗效进行客观、准确地评估，为临床治疗采取最佳方法提供依据。本文收集了近10年的随机对照研究（randomized controlled trials, RCT）中外文文献，通过采用*meta*分析的方法，比较评价完全电视胸腔镜手术与胸腔镜辅助小切口手术的治疗效果，以期更好地指导临床实践，并将经验推广交流。

## 1 资料和方法

### 1.1 文献检索

我们运用计算机检索PubMed、Embase、中国期刊全文数据库（CNKI）、维普期刊数据库（VIP）和ISI Web of Science。检索时间为2006年5月至2016年5月。收集国内外有关完全电视胸腔镜手术和胸腔镜辅助小切口手术治疗NSCLC的相关文章。检索CNKI、VIP等中文数据库的检索词采用“电视胸腔镜、胸腔镜辅助小切口手术、早期非小细胞肺癌”；检索PubMed、Embase等英文数据库的检索词为“Video-assisted thoracoscopic surgery（VATS），Video-assisted mini-thoracotomy（VAMT），Non-small cell lung cancer（NSCLC）”。

### 1.2 纳入和排除标准

纳入标准：研究类型：比较完全电视胸腔镜与胸腔镜辅助小切口手术治疗NSCLC的RCT研究。研究对象：确诊为原发性肺癌；患者的国籍、年龄、性别不限。干预措施：VATS与VAMT比较。结局指标：①有效性指标：淋巴结清扫数目；②安全性指标：手术时间、手术出血量、胸腔引流管放置时间、术后胸腔引流量、术后住院时间、手术死亡率、术后并发症发生率。排除标准：非RCT研究；缺乏相关临床研究数据。

### 1.3 资料提取

由两名研究人员独立评价文献并对符合纳入标准的文献进行提取资料，如遇意见不一致讨论解决或向第三方研究人员征求意见，尽量保证数据的准确性。

### 1.4 质量评价

依据CASP病例对照研究质量评价清单31.05.13版进行。对于CASP评价清单中的每条标准，如果参与评定的文献中明确满足者计2分；部分满足者计1分；不明确或未提及者计0分。评价过程由2位研究员独立实施，并进行交叉核对，如果有分歧则请第3位研究员解决或通过集体讨论解决。

### 1.5 统计学方法

运用Cochrane协作网提供的RevMan5.3软件进行*meta*分析。计量资料采用标准化均数差（standard mean difference, SMD）作为分析统计量；计数资料采用相对危险度（risk ratio, RR）作为分析统计量，各统计量区间估计采用95%可信区间（confidence interval, CI）表示，分析结果以绘制森林图来表示，发表偏倚检验结果以漏斗图来表示。对纳入的文献采用*χ*^2^检验进行异质性分析。若各研究间无明显异质性（*P* > 0.1, *I*^2^ < 50%），采用固定效应模型进行合并分析；反之则采用随机效应模型来进行分析^[5]^。

## 2 结果

### 2.1 文献检索结果

最初检索到文献103篇，通过阅读题目和摘要排除86篇重复文献和单臂研究，初步纳入研究17篇，阅读全文排除不符合纳入标准的研究4篇，最终纳入RCT 13篇^[6-18]^。文献检索流程见图 1。13项研究共1, 605例患者，VATS组815例，VAMT组790例，各研究均比较了患者年龄、性别等基线情况，结果显示两组间基线可比性较好（*P* > 0.05）。阅读纳入文献全文，提取数据资料及绘制表格。纳入研究的基本特征见表 1。

**1:**
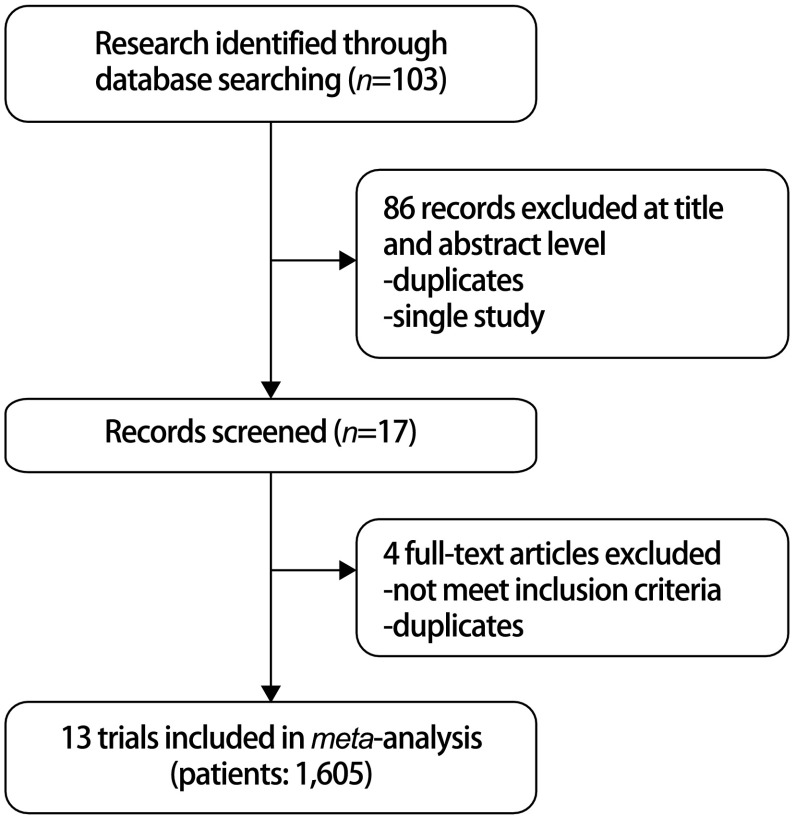
文献筛选流程图 Flow chart for study selection

**1:** 纳入文献的基本特征 Assessment characteristics of included studies

Included studies	Research year range	Case			Male/Female (*n*/*n*)			Age (year)			Tumor growth site (lung lobe)			
		VATS	VAMT		VATS	VAMT		VATS	VAMT		VATS		VAMT	
											Left	Right	Left	Right
Chu 2012^[5]^	2009-2011	162	221		87/75	119/102		57.7±10.0	59.6±11.3		60	102	85	136
Deng 2015^[6]^	2013-2014	32	44		20/12	27/17		62.03±7.87	63.44±8.02		14	18	19	25
Li 2015^[7]^	2012-2015	110	130		65/45	75/55		-	-		-	-	-	-
Li 2010^[8]^	2005-2007	34	35		-	-		-	-		-	-	-	-
Lu 2012^[9]^	2000-2010	24	26		17/7	16/10		69.7±6.3	68.1±7.6		9	15	10	16
Ma 2013^[10]^	2007-2010	39	56		24/15	36/20		62.34±8.45	63.40±9.51		18	21	26	30
Shi 2014^[11]^	2011-2013	30	30		-	-		15-76	15-76		-	-	-	-
Wei 2010^[12]^	2007-2010	23	31		14/9	18/13		60.2±6.2	61.7±5.9		10	13	14	17
Wu 2015^[13]^	2013-2014	31	31		-	-		45-75	45~75		-	-	-	-
Zhang 2014^[14]^	2009-2012	209	78		107/102	43/35		63.1±6.0	59.0±5.8		71	138	23	55
Zhang 2013^[15]^	2008-2013	47	43		30/17	26/17		62	63		15	32	15	28
Zhang 2009^[16]^	2007-2008	14	17		9/5	11/6		58.8±5.0	57.9±4.8		5	9	7	10
Zheng 2012^[17]^	2010-2011	60	48		37/23	32/16		52.1±12.8	53.1±11.6		26	34	23	25
VATS: video-assisted thoracoscopic surgery; VAMT: video-assisted minithoracotomy.														

### 2.2 文献质量评价

所纳入的文献均经CASP病例对照研究质量评价标准进行评分。纳入文献的基本情况见表 2。

**2:** 纳入研究的方法学质量评价 Assessment methodologic quality of included studies

Author	CASP
Chu 2012^[6]^	17
Deng 2015^[7]^	18
Li 2015^[8]^	14
Li 2010^[9]^	18
Lu 2012^[10]^	15
Ma 2013^[11]^	15
Shi 2014^[12]^	18
Wei 2010^[13]^	18
Wu 2015^[14]^	14
Zhang 2014^[15]^	16
Zhang 2013^[16]^	15
Zhang 2009^[17]^	16
Zheng 2012^[18]^	14

### 2.3 *Meta*分析结果

#### 2.3.1 两组手术安全性的的比较

##### 2.3.1.1 手术时间

纳入研究中，11项研究^[6, 8-10, 12-18]^报道了手术时间，两组共纳入患者1, 434例，其中VATS组744例，VAMT组690例。经*χ*^2^检验，各研究间存在异质性（*P* ＜ 0.000, 01, *I*^2^=88%），采用随机效应模型进行合并分析。结果（图 2A）显示：两组手术时间方面差异有统计学意义（SMD=13.56, 95%CI: 4.96-22.16），VATS组的手术时间长于VAMT组。

**2:**
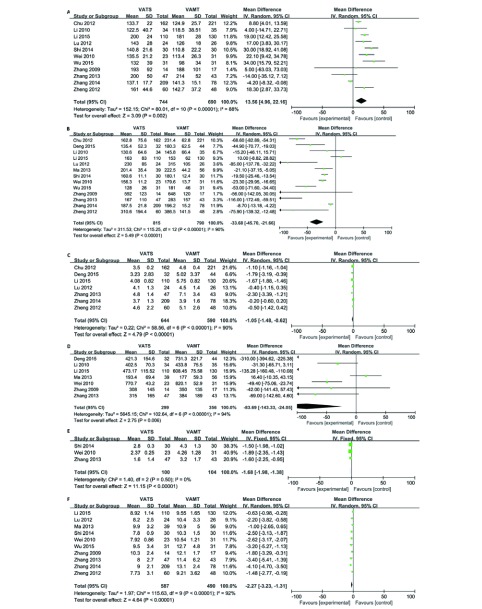
两组手术安全性的比较。A：手术时间；B：术出血量；C：置管时间；D：胸腔引流量；E：术后疼痛程度；F：术后住院时间。 The comparison of safety. A: the operating time; B: the intraoperative blood loss; C: chest tube placement time; D: chest tube drainage flow; E: postoperative pain scores; F: postoperative hospital stay.

##### 2.3.1.2 术中出血量

纳入研究中，13项研究^[6-18]^均报道了术中出血量，两组共纳入患者1, 605例，其中VATS组815例，VAMT组790例。经*χ*^2^检验，各研究间存在异质性（*P* ＜ 0.000, 01, *I*^2^=90%），采用随机效应模型进行合并分析。结果（图 2B）显示：两组手术出血量差异有统计学意义（SMD=-33.68, 95%CI: -45.70--21.66, *P* ＜ 0.000, 01）。表明VATS组手术出血量量较少。

##### 2.3.1.3 胸腔引流管放置时间

纳入研究中，7篇文献^[6-8, 10, 15, 16, 18]^报道了胸腔引流管放置时间。两组共纳入患者1, 234例，其中VATS组644例，开胸组590例。经*χ*^2^检验，各研究间存在异质性（*P* ＜ 0.000, 01, *I*^2^=90%），采用随机效应模型进行合并分析。结果（图 2C）显示：两组胸腔引流管放置时间差异有统计学意义（SMD=-1.05, 95%CI: -1.48--0.62, *P* ＜ 0.000, 01）。表明VATS组胸腔引流管放置时间较VAMT组短。

##### 2.3.1.4 术后胸腔引流量

纳入研究中，7篇文献^[7-9, 11, 16, 17]^报道了胸腔引流量。两组共纳入患者655例，其中VATS组299例，VAMT组356例。经*χ*^2^检验，各研究各研究间存在异质性（*P* ＜ 0.000, 01, *I*^2^=94%），采用随机效应模型进行合并分析。结果（图 2D）显示：两组胸腔引流管放置时间差异有统计学意义（SMD=-83.69, 95%CI: -144.33--24.05, *P*=0.006）。表明VATS组术后胸腔引流量较VAMT组少。

##### 2.3.1.5 术后疼痛程度

纳入研究中，3篇文献^[12, 13, 16]^报道了术后并发症发生率。两组共纳入患者204例，其中VATS组100例，VAMT组104例。经*χ*^2^检验，各研究间无统计学异质性（*P*=0.50, *I*^2^=0%），采用固定效应模型进行合并分析。结果（图 2E）显示：两组术后疼痛程度差异有统计学意义（SMD=-1.68, 95%CI: -1.98--1.38, *P* ＜ 0.000, 01）。表明VATS组术后疼痛程度较VAMT组更轻。

##### 2.3.1.6 术后住院时间

纳入研究中，10篇文献^[8, 10-18]^报道了术后住院时间。两组共纳入患者1, 077例，其中VATS组587例，VAMT组490例。各研究间存在异质性（*P* ＜ 0.000, 01, *I*^2^=92%），采用随机效应模型进行合并分析。结果（图 2F）显示：两组术后住院时间差异有统计学意义（SMD=-2.27, 95%CI: -3.23--1.31, *P* ＜ 0.000, 01）。表明VATS组术后住院时间较VAMT组短。

#### 2.3.2 两组手术效果的比较

##### 2.3.2.1 淋巴结清扫数目

纳入研究中共有9篇文献^[6, 7, 9, 10, 13, 15-18]^报道了清扫淋巴结数目情况。两组共纳入患者1, 148例，其中VATS组605例，开胸组543例。经*χ*^2^检验，各研究间没有异质性（*P*=0.14, *I*^2^=34%），采用固定效应模型进行合并分析。结果（图 3A）显示：两组淋巴结清扫数目差异有统计学意（SMD=-0.48, 95%CI: -0.80--0.17, *P*=0.003）。表明VATS组手术清扫淋巴结数目较VAMT组多。

**3:**
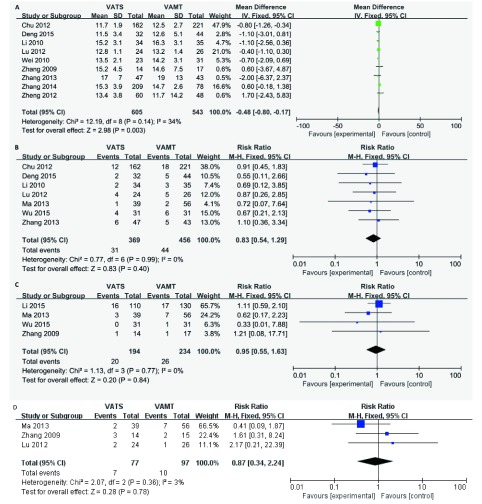
两组手术效果的比较。A：术中淋巴结清扫的个数；B：术后并发症；C：手术死亡率；D：复发率。 The comparison of efficacy. A: harvested lymph nodes; B: postoperative complications; C: operation mortality; D: recurrences.

##### 2.3.2.2 术后并发症发生率

纳入研究中，7篇文献^[6, 7, 9-11, 14, 16]^报道了术后并发症发生率。两组共纳入患者825例，其中VATS组369例，VAMT组456例。经*χ*^2^检验，各研究间无统计学异质性（*P*=0.99, *I*^2^=0%），采用固定效应模型进行合并分析。结果（图 3B）显示：两组术后并发症发生率差异无统计学意义（RR=0.83, 95%CI: 0.54-1.29, *P*=0.40）。表明两组术后并发症发生率相当。

##### 2.3.2.3 手术死亡率

纳入研究中，4篇文献^[8, 11, 14, 17]^报道了手术死亡率。两组共纳入患者428例，其中VATS组194例，VAMT组234例。经*χ*^2^检验，各研究间无统计学异质性（*P*=0.77, *I*^2^=0%），采用固定效应模型进行合并分析。结果（图 3C）显示：两组手术死亡率差异无统计学意义（RR=0.95, 95%CI: 0.55-1.63, *P*=0.84）。表明两组手术死亡率相当。

##### 2.3.2.4 1年复发率

纳入研究中，3篇文献^[9, 10, 16]^报道了近1年复发率。两组共纳入患者174例，其中VATS组77例，VAMT组97例。经*χ*^2^检验，各研究间无统计学异质性（*P*=0.36, *I*^2^=3%），采用固定效应模型进行合并分析。结果（图 3D）显示：两组手术死亡率差异无统计学意义（RR=0.87, 95%CI: 0.34-2.24, *P*=0.78）。表明两组手术年复发率相当。

### 2.4 发表偏倚分析

对纳入的研究，通过绘制漏斗图来分析发表偏倚，见图 4。该图呈现出了较好的对称性，说明发表偏倚的可能性较小。

**4:**
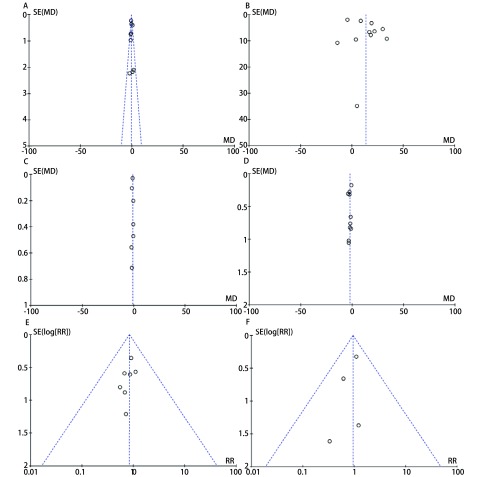
发表偏倚漏斗图。A：淋巴结清扫数目；B：手术时间；C：胸腔引流管留置时间；D：术后住院时间；E：术后并发症发生率；F：手术死亡率。 Funnel plot of publication bias. A: number of lymph node dissection; B: operation time; C: chest tube placement time; D: post-operation hospital stay; E: postoperative complications; F: operation mortality.

## 3 讨论

NSCLC治疗手段仍是手术切除为主。传统的手术手术切口长，出血多，术后疼痛明显并且恢复慢，并伴随着高并发症^[19]^。VATS于上世纪90年代应用于临床，2006年美国国家综合癌症网（National Comprehensive Cancer Network, NCCN）公布的治疗指南将VATS作为早期NSCLC的标准治疗方式^[20-22]^。与传统开胸手术相比，胸腔镜外科手术创伤小、疼痛轻、恢复快的微创优势已经得到证实，但是，开展全VATS（c-VATS）难度大、学习周期长^[22]^。由于经济条件的差异等诸多因素影响，国内大多数是各种类型的VAMT，真正意义上的VATS仅限于少数。虽有部分学者对二者的临床疗效进行了比较，但得出的临床结论各有差异。参照2012年发表在中国肺癌杂志上的胸腔镜与开胸肺叶切除术治疗NSCLC近期疗效的系统评价。本文收集了13项RCT对比了VATS和VAMT在术中淋巴结清扫个数、手术时间、术中出血量、引流管放置时间、术后胸腔引流量、术后住院时间、并发症发生率及手术死亡率进行了*meta*分析，旨在对两者治疗NSCLC的近期疗效做出系统评价。

本研究存在一定的局限性：①尽管尽可能全面地收集近10年的文献，但由于收集到的文献语种主要为中文，难以避免会存在一定的偏倚；②纳入文献的质量高低不等，部分文献对随机方法、是否采用盲法等情况都未作详细叙述；③纳入文献的部分效应指标存在较大异质性，原因可能是纳入的研究均为回顾性临床对照研究，在实验设计、实施过程中缺乏科学性和严谨性，对分析结果也有一定的影响。因此，仍需更多、质量更好的随机对照研究对比两组手术治疗NSCLC的临床疗效，从而得出更有说服力的结论。

综上所述，通过对纳入的研究进行*meta*分析，可以认为完全VATS与VAMT治疗非NSCLC在淋术后并发症的发生率和手术死亡率方面比较疗效相当；在缩短住院时间方面，VAMT有一定的优势；但在减少手术出血量、胸腔引流量、胸腔引流管的放置时间以及术后疼痛感和住院时间方面，完全VATS优势明显。本研究结果提示完全电视胸腔镜行肺癌根治术是目前值得推广的治疗NSCLC的手术方式。此外文献还报道VATS对免疫系统干扰小，患者恢复快，能够及时接受化疗等术后辅助治疗以及能够更好地耐受全疗程化疗^[23]^；对呼吸功能影响小；手术视野的暴露、病变细微结构的显像、手术切除范围的判断及安全性好、对凝血功能影响小、血栓发生率低等优点^[24]^。在VATS开展初期，对所有患者都进行胸腔镜探查，在VATS中合理掌握中转VAMT的指征，经过一段时间的训练，有可能在肺手术总量不大的医疗机构较为安全地掌握和开展VATS。
